# End-of-life management strategies of pharmaceuticals in Portuguese households

**DOI:** 10.1177/0734242X221105416

**Published:** 2022-07-27

**Authors:** Anabela Veiga, Ana Catarina Sousa, Cláudia Sousa, Manuel Oliveira, Belmira Neto

**Affiliations:** 1 LEPABE – Laboratory for Process Engineering, Environment, Biotechnology and Energy, Department of Chemical Engineering, Faculty of Engineering, University of Porto, Porto, Portugal; 2 ALiCE – Associate Laboratory in Chemical Engineering, Faculty of Engineering, University of Porto, Porto, Portugal; 3 Universidade Católica Portuguesa, CBQF – Centro de Biotecnologia e Química Fina – Laboratório Associado, Escola Superior de Biotecnologia, Porto, Portugal; 4 Departamento de Clínicas Veterinárias, Instituto de Ciências Biomédicas de Abel Salazar, Universidade do Porto, Porto, Portugal; 5 Centro de Estudos de Ciência Animal, Instituto de Ciências, Tecnologias e Agroambiente da Universidade do Porto (ICETA), Porto, Portugal; 6 Accenture Technology Center Braga, Braga, Portugal; 7 DEMM, Faculty of Engineering, University of Porto, Porto, Portugal

**Keywords:** Disposal, end of use, management, pharmaceutical products, survey, waste

## Abstract

The end of life of pharmaceutical products through environmentally unsafe routes is a growing concern in our society. However, the studies reported so far, apart from being limited in number, do not reflect the current management practices for the end-of-life management of pharmaceuticals. Published work lacks an in-depth analysis in focusing on the pharmaceutical waste in households. The present work focused on (1) performing a state-of-the art overview to compare worldwide studies and the results and (2) implementing a comprehensive survey in Portugal (*n* = 454 respondents). The results showed that the delivery to pharmacies was used by the majority of the respondents (72%), indicating a good awareness of pharmaceutical waste management issues, when compared to the reviewed studies. Statistically significant variables for the destination of end-of-use pharmaceuticals include gender, age and distance from the residence to the pharmacy (*p* < 0.05). Most participants believe that educating the population on existing structures of medication and packaging management is of the utmost importance to improve the national managing system. This is the first study conducted in Portugal; it includes statistical analysis of the data and reflects on the practices that should be adopted to reduce incorrect pharmaceutical waste disposal. These findings call upon the strategies to strengthen the pharmaceutical waste management programme.

## Introduction

### Destinations of pharmaceutical wastes in households

Pharmaceutical products, also known as medicines or drugs, have an unquestionable relevance in our society, helping to fight diseases and increasing the quality of life and its longevity (Sofia and Santos, 2014). In the last decades, pharmaceutical consumption has increased, partly due to the increase in the need for drugs to treat ageing-related and chronic diseases and due to the changes in clinical practices (OECD, 2017). In fact, according to a market research, the global pharmaceutical market was worth $934.8 billion in 2017 and is expected to reach $1170 billion in 2021. This increase is estimated to contribute to an annual growth rate of over 5.8% (The Business Research Company, 2018). Although pharmaceutical products are fundamental to both modern and traditional medicine, scientists, regulatory agencies and the European Commission (EU) have acknowledged that these products are contributing to emerging environmental problems due essentially to the end of life of unused and expired medicines used in households (Küster and Adler, 2014). The Directive 2013/39/EU plays an important role in limiting the water pollution caused by pharmaceutical substances in an aquatic media (The European Parliament and the Council of the European Union, 2013). The most recent strategy to tackle this problem aims to counteract the negative effects of pharmaceuticals on the environment, covering their whole lifecycle from design and production to disposal (European Commission, 2020; Lima et al., 2020).

**Figure 1.**
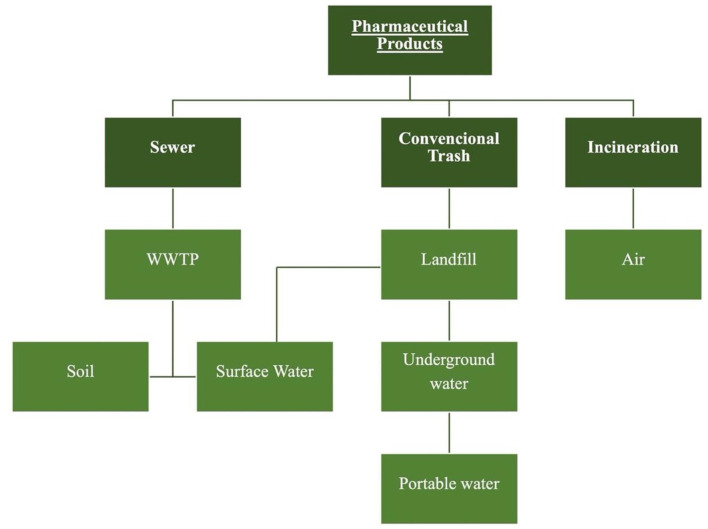
End-of-life destinations for unused pharmaceutical products by households. Source: Dębska et al. (2010), Bound and Voulvoulis (2005) and Shipingana et al. (2022). WWTP: wastewater treatment plant.

Some studies conclude that a large contribution to the environmental impacts of pharmaceuticals occurs at the consumer level, as they dispose the pharmaceutical wastes in sinks, toilets and waste bins. The incorrect disposal of drugs may harm the ecosystem and the world population, causing ecotoxicity and estrogenic activity (e.g. hormonal dysfunction and dysregulation) (Jos et al., 2006; Tong et al., 2011). Figure 1 shows the potential contamination of soil, water and air associated with the destination for pharmaceutical waste.

Unused pharmaceutical products flushed into sinks and toilets reach wastewater treatment plants (Patel et al., 2022). These are mostly inefficient in removing these contaminants from wastewaters and as a result vestiges of unwanted pharmaceuticals are present in the treated wastewater streams (Bound and Voulvoulis, 2005; Boxall, 2004; Salgado et al., 2010). When pharmaceutical active compounds are discarded in household trash bins and get deposited in landfills, environmental contaminations can occur. Consequently, it results in pollution of groundwaters due to landfill leachates. Several types of pharmaceuticals, such as antibiotic, antipyretic, antiepileptic, antipsychotic drug residues (González-González et al., 2022; Kanama et al., 2018), have already been found in the environment at relatively low concentrations (µg/L, ng/L), but these compounds have high bioavailability and pharmacological potency, which may lead to severe environmental problems (Nunes, 2011; Pandis et al., 2022).

Current studies that focus on antibiotic resistance often indicate an increase in resistance genes but give little information about the associated risks of transmission, and thus the potential impact on human health (Kraemer et al., 2019; Sharma et al., 2022). Antipyretic drugs cause acute toxicity only at high concentrations, whereas sub-lethal effects at low, environmentally relevant concentrations (adverse effects at molecular, biochemical and cellular levels) (Parolini, 2020). Antiepileptic and antipyretic drugs at low concentration also affect living organisms by acting on the specific biochemical pathways that are evolutionarily conserved (Martin-Diaz et al., 2009; Kalichak et al., 2017).

Pharmaceutical wastes can also be disposed by incineration and it may seem to be a preferable route for the end of life. However, environmental contamination occurs due to the release of toxic air pollutants (Amaral and Fop, 2013). Pharmaceutical waste incinerators emit toxic air pollutants and toxic ash residues that are the major source of dioxins in the environment and can cause cancer, reproductive and developmental problems, damage to the immune system and can interfere with hormones (Gautam et al., 2010).

### The Portuguese reality

Due to the potential relevant environmental problems caused by the end of life of pharmaceuticals, many countries have implemented collecting systems for pharmaceutical waste generated within households. In Continental Portugal and islands, VALORMED is the entity responsible for the waste management of pharmaceutical products including the management of its packaging waste. The main goal of this non-profit company is to provide an autonomous system for the collection and treatment of medicines in their end of life, resulting from the pharmaceutical industry, hospitals and veterinarians, which undergo a safe collection and waste valorisation process (Decree-Law No. 152-D/2017). After being collected by VALORMED, the pharmaceutical wastes are separated and classified so that they can go through an appropriate valorisation system comprehending recycling of paper, cardboard, plastic and glass and incineration with energy recovery of the remaining residues, namely, remnants of medicines. Thus, VALORMER strategic approach comprises the structuring of a network of selective collection, financing the costs of sorting, storage, transport, treatment and recovery of packaging waste deposited in the selective collection networks, and the compliance with collection targets and minimum recovery objectives (VALORMED, 2019a).

However, the total amounts of unused pharmaceuticals in the households, which are not sent for valorisation by VALORMED, are unknown, which is in line with other countries. According to the study of Vogler et al. (2014), although the information regarding the amount of medicines that are not discarded properly is lacking, it is estimated that 10% of sold medicines end up in household waste. Moreover, a review of existing literature concluded that measurements of the quantity of medicines discarded in proportion to total sold are not available and that the best estimates identify a range of 5–10% (Castensson and Ekedahl, 2010).

According to VALORMED, the number of collected pharmaceuticals and materials used in its packaging has increased over the last few years (VALORMED, 2019b). In 2019, 5 million tonnes of municipal waste were produced in Portugal (REA, 2021). Of these, approximately 1216 tonnes were deposited by citizens at collection points located in national community pharmacies, corresponding to an average of 109 g per inhabitant, resulting in an increase of 9% over the previous year (VALORMED, 2019b). Nevertheless, a national study, which inquired a total of 243 households, states that approximately a third of the families do not return household pharmaceutical waste to pharmacies preventing them to undergo adequate valorisation routes (Dias-Ferreira et al., 2016). However, this study was conducted in 2016 and no new scientific evidence is available about the practices adopted by the Portuguese households. This is particularly important in the last decade, where there has been an increased environmental awareness and a greater effort by policymakers to adopt appropriate pharmaceutical disposal practices (OECD G20, 2021).

Prevention practices, which are supported by the Portuguese government, include consumer awareness strategies, guidance from health professionals, guidance by pharmacies, guidance through information on medicine packaging and package leaflets, and guidance through advertising campaigns announcing the strategies and practices for the procedures for returning medicine wastes (Magalhães, 2020).

### Relevant international studies

Several studies have been conducted to identify and characterize the common practices of citizens from worldwide countries concerning the disposal of unused pharmaceuticals from households (Dias-Ferreira et al., 2016; Glassmeyer et al., 2009; Insani et al., 2020; Kahsay et al., 2020; Rogowska et al., 2019). Most investigations also evaluate the perception of the environmental and health risks stemming from inappropriate disposals. Table 1 lists a total of 14 studies, from different countries across the world, based on the research carried out between September 2020 and March 2021 with the following keywords: end-of-used/unused/expired pharmaceuticals/drugs, disposal and management practices. The searching engines are Google Scholar, Science Direct (Elsevier), Scientific Electronic Library Online (SciELO) and Springer Link. A set of studies, held between 2005 and 2020, were selected and reviewed. In addition, only studies written in English, in which a survey was implemented, were considered. Studies had to survey a minimum population of 150 respondents and the keywords ‘disposal practices, expired/end-of-life pharmaceuticals’ were considered as inclusion criteria. The studies that do not fulfil these keywords were excluded from the subsequent analysis.

**Table 1.** Set of relevant reviewed studies on pharmaceutical household waste practices.

Region	Year	Survey theme and objectives	Sampling	Medication disposal practice	Knowledge regarding the established practices	Other relevant observations	References
Bandung, Indonesia	2020	To evaluate the disposal practices of unused and expired medicines among the general population	475 respondents	- The most common disposal method of unwanted medicines was throwing away in household garbage (82.1%).	- A significant percentage of the respondents never received information about proper medication disposal practice (79.5%); - More than half of the respondents were unaware that unsafe medication disposal practices could harm the environment and population health (53.1%).	- The majority of the respondents checked the expiration date of the drugs before purchasing (72.8%); - Approximately 95% of the respondents had unused medicines stored in their homes, with NSAIDs, vitamins/nutritional supplements and antibiotics were the most common types of medicines left unused.	Insani et al., 2020
Adigrat City, Northern Ethiopia	2020	To assess knowledge, attitude and disposal practice of unused and expired pharmaceuticals in the community.	359 respondents	- Around three-quarters (75.2%) and 63% of the respondents discarded unused and expired medicines in the garbage bins, respectively.	- Almost half of the respondents (50.14%) have good knowledge concerning the disposal of unused and expired pharmaceuticals.	Most (82.2%) of the respondents have a positive attitude towards the disposal of unused and expired pharmaceuticals. - Around 52 (52.4) of the respondents had unused medicines stored at home, with analgesics being the most common (41.5%).	Kahsay et al., 2020
Poland	2019	Survey I – Identify the scale of pharmaceutical consumption and the way pharmaceuticals are disposed of by various social groups; Survey II – Identify patient’s attitudes regarding expired/unused pharmaceuticals at home.	Survey I – 450 people Survey II – 635 people	- Almost 68% usually disposed of expired pharmaceuticals along with their household waste or via the toilet into the sewage system.	- Most respondents (over 65%) who participated Survey I indicated that they were aware that pharmaceutical waste can be returned to pharmacies.	- The most commonly used over-the-counter drugs included analgesics (71.8%), cold and flu drugs (65%) and vitamins and minerals (41.3% of respondents).	Rogowska et al., 2019
Hong Kong	2019	- Evaluate the practices in handling and disposing unused pharmaceuticals in the household.	1865 respondents	- Residents most frequently dispose of unused medicines in regular waste bins (53.9%).	- 62.3% of respondents strongly disagreed or disagreed that disposing unwanted medicines in waste bins, through drains or in toilets would not significantly harm the environment.	- 75% indicated that at the time of the survey they had unused drugs at home. - Normal consumption of unused medicines following self-diagnosis of symptoms.	Chung and Brooks, 2019
District of Nicosia (Cyprus)	2018	- Evaluation of the attitude of citizens regarding the disposal of pharmaceuticals as well as to identify the main reasons why pharmaceutical wastes are produced.	184 individuals	- Most favourable disposal practice of unused and expired drugs at the household level from citizens includes waste garbage (92.40%) and toilets/sink (24.50%).	- The absence of a specific management plan is considered to be an issue. The implementation of prevention activities and public awareness event regarding the negative impact on the environment of the uncontrolled disposal of the pharmaceutical waste, negative issues to the health that are created using medicines and drugs without doctor’s advice and safe disposal methods of waste will be very useful for the population and for the protection of the environment.	- Painkillers were the most used by the respondents (65.8%); - 86.6% of men and 83.3% of women used the pharmacy with or without a doctor’s prescription.	Zorpas et al., 2018
Saudi Arabia	2018	- Evaluate the perception of environmental and health risk associated with pharmaceuticals and to investigate the factors influencing the choice of disposal methods.	767 respondents	- Majority of respondents (62.9%) discarded unwanted medications in household waste, with the remainder emptying them into the sink or toilet (16.6%), returning them to a pharmacy (6.5%) or a physician (1.4%).	- Of the respondents, 73% declared that they had never received any instructions about proper ways of disposing medications.	- Respondents possessing higher level of education and people who received instructions regarding proper disposal are more likely to return pharmaceutical waste to a pharmacy.	Shaaban et al., 2018
Accra (Ghana)	2017	The objective of this study was to investigate the household disposal practices and harm resulting from solid medical waste generated in households and the community.	600 households	- 80% and 89% of respondents discarded unwanted medicines and sharps in household refuse bins, respectively.	- Knowledge about disposal methods could be helpful in accounting for possible losses in the estimates of solid medical waste generated from residential dustbins.	- Of the people who discarded medicine in bins, 23 and 35% of respondents discarded these items without a container. Harm from solid medical waste in the household and in the community was reported by 5 and 3% of respondents, respectively.	Udofia et al., 2017
Kabul	2017	- Report the current practices and attitudes of general public towards disposal of unused and expired pharmaceuticals.	301 individuals	77.7% of the respondents discarded the expired medicines in household trash.	- Majority of respondents felt that government should take the responsibility for creation of awareness for proper medicine disposal. Almost entire sample (98%) felt that improper disposal of unused and expired medicines can affect the environment and health.	- 83.4% of the interviewees purchased medicines on the prescription; - Among the respondents, 46.5% purchased antibiotics and the remaining purchased NSAIDs, anti-hypertensive and anti-diabetic medicines; - Majority (95.3%) of the respondents stored medicines at home.	Bashaar et al., 2017
Portugal	2016	- Provide a first insight of the citizen’s awareness and daily practices concerning household pharmaceutical waste, with a strong focus on disposal pathways.	244 families	Face-to-face interviews with householders showed that 69% of the respondents claimed returning pharmaceutical waste to the local pharmacy.	- People tended to link discarding household pharmaceutical waste via the wastewater with increased risk for the environment; - Discarding household pharmaceutical waste into the waste bin was also perceived as hazardous for the environment.	- On average, in each household, 20% of pharmacological products were in use, 72% were not in use and 8% were mostly expired products ready to discard.	Dias-Ferreira et al., 2016
Serbia	2010	- Investigate the storage and disposal habits of medications among the population.	208 families	- The most common method for disposal of household medications is disposal in the garbage (85.6% (urban) and 74.5% (rural)) or in the toilet (8.7% (urban) and 6.4% (rural)); - 4% of respondents leave unused pharmaceuticals at pharmacies.	- Despite the fact that one-half of the population is aware of the harmful effects of medications on the environment, most of the population still dispose unused medications in a way that can have serious consequences on the environment.	- Drugs were mostly kept at home in a specific place-home pharmacy (89.8% (urban) and 89.0% (rural)); - The frequency of expired medications was not observed to be different between the urban and rural households (10.3% (urban) and 11.8% (rural)).	Kusturica et al., 2012
Sweden	2009	- This study aimed at learning about what the general public does with unused prescription drugs as well as the pertaining attitudes to this issue.	1000 responders nationwide via telephone (from 2001 to 2007)	- Pharmaceuticals are disposed of with solid waste only by 3% of households and 43% of households return them to pharmacies.	- 85% knew that correct disposal was to return unused medicines to a pharmacy; - 50% answered that they returned the unused medicines for environmental reasons and 42% answered that they worry about the environmental impact of medicines.	- 43% had in fact returned their medicines to a pharmacy during the last 12 months.	Persson et al., 2009
New Zealand	2009	- The aim of this study was to determine the proportion of unused medications that are not returned to a pharmacy for disposal and are instead disposed of via land fill or water systems.	452 individuals	- Depending on formulation type, between 13 and 24% of unused medications were returned to pharmacies with tablets and capsules being most likely to be returned and liquids most likely to be added to water systems (this percentage depends on the formulation type).	- One-half of the population is aware of the harmful effects of medications on the environment; however, most of the population still dispose unused medications in a way that can have serious consequences on the environment.	- 62% of respondents currently had unwanted medications in their house.	Braund et al., 2009
United States	2006	- Identify patients’ disposal habits and explore patient’s beliefs about disposal methods.	301 patients	- Only 22.9% reported returning medication to a pharmacy for disposal; - More than half had flushed them down a toilet.	- Less than 20% had ever been given advice about medication disposal by a healthcare provider.	- More than half of the patients surveyed reported storing unused and expired medications in their homes.	Bound et al., 2006
United Kingdom	2005	- This study aimed to investigate the link between risk perception and household disposal.	392 people	- 63.2% of unused medicines are disposed of with the solid waste and 21.8% are returned to pharmacies.	–	- Although half of the people finished their prescriptions, reasons for disposal included expiration (30.7%) and completion of treatment before finishing the prescription (12.2%).	Bound and Voulvoulis, 2005

These studies were based on the responses via telephone, face-to-face interviews and surveys with the main goal of evaluating disposal practices, enabling to establish a correlation between these practices and the social, demographic and economic factors. Most questionnaires address the same issues for assessing the pharmaceutical disposal practices which include the most common disposal practices used by households, the perception of the associated environmental risk, and socioeconomic and geographical factors.

Findings show that the remote and anonymous surveys are considered the best approach to obtain more accurate answers (Ariffin and Zakili, 2019). On one hand, anonymity ensures honest feedback. On the other hand, straightforward questions that can be answered in a short time remotely are more likely to be answered (Murdoch et al., 2014).

The most common practice for the disposal of household medications, mentioned by the largest number of the inquired respondents, is discarding the waste into the trash bin or into the toilet/sink. Thus, it is possible to infer that there is still a lot of work to be done to inform worldwide citizens about the improper disposing of the unused pharmaceuticals causes harmful effects in the environment. However, in recent studies, more public awareness and more robust assessment practices are shown, which means that the inclusion of issues related to education/knowledge of the practices adopted has been increasing and being explored in works of this nature (Table 1).

In fact, in the works carried out in the last 4 years, the majority of the population surveyed stated to be acquainted that the incorrect disposal of pharmaceutical waste products leads to environmental damages. These studies also address the aspects such as compliance to medication instruction, attitudes towards best source of awareness and the role of a community pharmacy (Chung and Brooks, 2019; Insani et al., 2020; Kahsay et al., 2020; Rogowska et al., 2019).

Another finding relates to the fact that despite the growth in the research on this topic, the review shows that most of the studies lack on a more global analysis of the problem associated with the improper disposal of pharmaceutical wastes from households. This is to say that a lot still needs to be done in the analysis of the reality of the country in which the studies should focus to compare it with the studies conducted worldwide. In this research field, it is crucial to conduct a state-of-the-art revision to understand what the most pertinent questions are to include in the survey, as well as to compare the findings with the work from different countries. This allows us to understand what can be improved, to seek solutions and alternatives that are succeeding in other geographical contexts. The review also shows that statistical analysis is almost never used in these studies to evaluate which variables affect the answers reported by the respondents.

This work presents an overview on the pharmaceutical waste generated across the world (Table 1). Moreover, the present study is the second study in Portugal on the topic of wasted medicines, and being the first study with a complete analysis of the social, demographic and economic factors that may influence the practices regarding the end-of-use pharmaceuticals based on statistical analysis. The first nationwide study in Portugal was conducted in 2016. However, as previously stated, more recent data are essential due to the relevancy of this environmental problem in our society. Such analysis aims to provide an insight national on current practices for the end of life of unused medicines at home to assist on giving solid arguments to a more effective management by the national agents responsible for the valorisation of such medicines.

## Materials and methods

### Survey characteristics and objectives

The survey was designed and conducted using online survey tools from Google Forms (www.google.com/forms). The questionnaire was initially piloted using a group of nine random people, in order to check unambiguity of questions and to test the fact that instructions were correctly interpreted by the participants. The survey was conducted through email, social media platforms (disseminated in groups related to the environment and health) and network at the University of Porto and VALORMED site. The questionnaire was available for 1 year, between February 2019 and February 2020. The survey questions were formulated envisaging the goals presented in Table 2. The full survey is presented in the Supplemental Information (Survey S1).

**Table 2.** Survey questions and related main goals.

Survey questions topics	Main goal
Gender, age, education, occupation, marital status, residence and household	Relate the personal data and the distance from the residence to the pharmacy with the way the population dispose the medicines.
Distance from the residence to the pharmacy	
Number of different medicinal packages at home	To analyse the number and type of medicines at home of the Portuguese population, given the fact that they may have distinct effects in the environment.
Type of medicines at home	
How medicines are being disposed	Typify and quantifying the population that disposes medicines in pharmacies
In which situations, the medication is disposed of and who is doing it	Identify the person of the household who normally discards the medications and understand the choice of the option for the disposal.
Time length since the last destination to medicines out of use	
The main reason for disposing of medicines out of use	
Overview the current system of delivery of medicines out of use in pharmacies	Analyse the population’s opinion and identify the new measures to increase the delivery to disposal in pharmacies.
Overview different measures to sensitize the correct management of medications at the end of use	

Statistical analysis was performed using Excel (version 14.0.7266.5000). Comparisons between groups were performed by chi-square independence test. Dependency was considered statistically significant when *p* < 0.05 (95% confidence). This indicates a strong evidence against the null hypothesis, as there is less than a 5% probability the null is correct (and the results are random). In these scenarios, the variable under study is proven to have influence in the pharmaceutical discard practices of Portuguese households.

Significance results are also indicated according to *p* values with one, two, three or four of the symbols (*) corresponding to 0.01 < *p* ⩽ 0.05, 0.001 < *p* ⩽ 0.01, 0.0001 < *p* ⩽ 0.001 and *p* ⩽ 0.0001, respectively. This methodology was applied to evaluate whether the gender, age, education, professional situation and distance from the residence to the pharmacy had any influence on the disposal practices adopted. Boxplots were also used graphically to represent the groups of numerical data through their quartiles. This methodology was applied to analyse the population opinion concerning the presented measures in order to identify where the focus of action should aim to improve the practices in Portugal and serve as a platform for other countries.

## Results and discussion

### Characterisation of the population of responders

**Figure 2.**
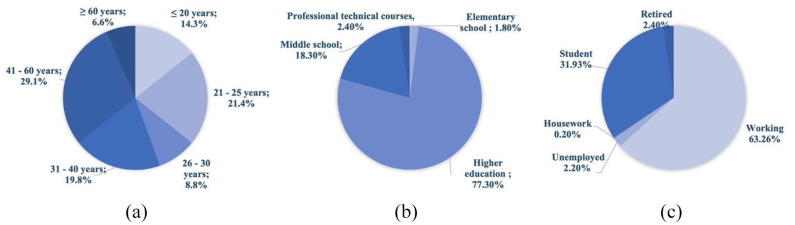
Characteristics of respondents: (a) age, (b) level of education and (c) current occupation.

The population sample consisted of 454 individuals, including 156 men (34.4%) and 298 women (65.6%) (Supplemental Figure S2.1a). The majority of participants are aged between 41–60 years (29.1%) and 21–25 years (21.4%). These age ranges can be explained in the forms of dissemination of the questionnaire used, since social media platforms are currently mostly used by adults and college institutional emails are used by younger students (Figure 2(a)). The educational level of the respondents is as follows: 77% have superior education, 18% have high school and 2.4% have professional/technical courses (Figure 2(b)). Most respondents are currently working (63%) or are students (32%) (Figure 2(c)).

Although the sample of the present study is from several districts in Portugal, the majority lives in the north, mainly in the district of Oporto, Portugal (72.2%) (Supplemental Figure S2.2). Approximately 17.5% of the Portuguese population live in this city, and it is not possible to associate the respondents’ area of residence with their drug disposal practices, since we do not have a significant sample for the remaining cities. Thus, the characteristics of the respondents (the majority being women, with higher education and living in Oporto) is the limitation of this study as it may not be a total representative of the Portuguese population. However, the statistical analysis performed is indeed complete in considering the whole set of the answers received. Moreover, as far as the authors’ knowledge, it is the first study in this topic.

### Quantity and type of drugs in Portuguese households

Responses on the quantity of different pharmaceuticals and packages that families keep in their households showed that about 31% indicated a number between 11 and 20 and 23% between 21 and 30% (Supplemental Figure S2.4a). This is similar to the results obtained in different countries, where most respondents have unused medicines stored at home (Bashaar et al., 2017; Braund et al., 2009; Rogowska et al., 2019). According to Ferreira and co-authors (Dias-Ferreira et al., 2016), in Portuguese households, approximately 70% are pharmaceutical products that are not being used.

Some authors state that the large amount of medication that families have at home can be explained by a trend that has been observed worldwide, which is the purchase of pharmaceutical products without a prescription (Chung and Brooks, 2019; Zorpas et al., 2018). Another reason may also be related to the fact that the number of pharmaceuticals in a package is superior to what is indispensable for the patient treatment.

**Figure 3.**
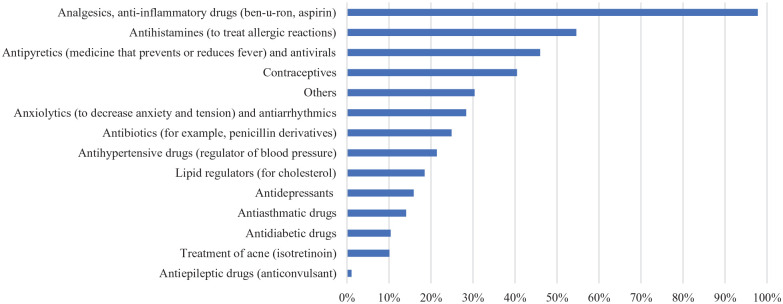
Type and frequency reported on pharmaceutical products at home.

Regarding the type of pharmaceuticals, almost all families have anti-inflammatory (98%) drugs and approximately a half have antihistamines and antipyretics (Figure 3). Anti-inflammatory drugs are the most frequent medicines that families keep in their home (Insani et al., 2020; Kahsay et al., 2020; Rogowska et al., 2019; Zorpas et al., 2018). The hazardous effects that these pharmaceutical products have in the environment are known. Recent research has suggested that ibuprofen (a commonly used analgesic) poses an unacceptable risk of about 50% of river that reaches across 22 catchments in Britain (Warhurst, 2017). In studies on the effects on fish reproduction, ibuprofen was reported to cause male fish to abnormally make the female egg yolk protein, vitellogenin and parental exposure to levels as low as 0.0001 mg/L Ibuprofen® delayed the hatching of eggs, an effect which can increase the risk of predation. Moreover, Diclofenac®, an anti-inflammatory, caused the death of thousands of vultures between 1996 and 2007 in Asia and affected fish and other wildlife species in other countries (Warhurst, 2017). Other pharmaceutical products at home can include hormonal preparations, anti-infective drugs and vitamins (Drugs.com, n.d.).

### Analysis of disposal pathways

The survey showed that approximately half of the respondents do not separate the packaging from the medication (around 51%), while the other half performs the separation (Supplemental Figure S2.4b). This is particularly relevant in Portugal, since as previously explained, VALORMED collects both medicinal and packaging wastes. However, VALORMED only quantifies the total amount of waste collected. Therefore, there is no differentiation between medicinal and their packaging wastes (VALORMED, 2019b).

Results show that the destination that people normally give to unused or expired pharmaceuticals can be divided into several paths: the delivery to the pharmacy (72%), deposit in the household waste bin (30%), deposit in the sewer/sink/toilet (about 2%) or others (5%). Thus, most of the respondents state that they deliver it to the pharmacy. In a previous study conducted in Portugal, although 69% of the respondents claimed returning pharmaceutical waste to the local pharmacy, the authors argue that this high value can result from the type of methodology followed: face-to-face questionaries can have a ‘good answer effect’ (Dias-Ferreira et al., 2016). The results obtained in the present work are in line with the ones of Dias-Ferreira et al. (2016), and it showed that this is not the case.

In comparison with other countries, where disposal through garbage and sewage are the most commonly used routes, Portugal appears to adopt better pharmaceutical management practices (Table 3). These disposal practices are more similar to those found in works conducted in Sweden and Australia (Insani et al., 2020). Among those who deliver to the pharmacy, the majority (around 72%) discard the pharmaceuticals in the moment they visit the pharmacy. On the other hand, around 28% of respondents go to the pharmacy purposely to deliver the unused pharmaceuticals (Supplemental Figure S2.5b). It was also possible to verify that within the family, the person responsible for discarding medicines out of use in the pharmacy is usually the mother (occurring in 52% of the cases) (Supplemental Figure S2.6a).

**Table 3.** Independency review (χ2) between the demographic and social characteristics on the destination of end-of-use pharmaceuticals.

Destination to end-of-use pharmaceuticals	Gender		Age		Education		Professional situation		Distance from the residence to the pharmacy	
	χ^2^	*p*-Value	χ^2^	*p*-Value	χ^2^	*p*-Value	χ^2^	*p*-Value	χ^2^	*p*-Value
(1) Pharmacy										
(2) Sewer/bin/toilet										
(3) Household waste										
(4) Other										
(5) Pharmacy and Sewer/bin/toilet										
(6) Pharmacy and household waste	21.5	0.01*	70.4	0.009*	16.4	0.99	32.5	0.63	84.78	6.96 $\times 10^{-8}$ ****
(7) Pharmacy and others										
(8) Sewer/bin/toilet and household waste										
(9) Household waste and others										
(10) Pharmacy/household waste and others										

The relationship is significant at *0.01<*p* ⩽ 0.05, ****p ⩽ 0.0001.

In addition, around 74% of people stated to discard the pharmaceutical products in the last year (Supplemental Figure S2.6b). The regularity of delivery of end-of-use drugs is also comparable with the study conducted in Sweden, where approximately 40% of the respondents had returned their medicines to the pharmacy during the last year (Persson et al., 2009).

The other findings suggest that the main argument that leads to the disposal of unused medication is the expiration date (86%). This is complemented by other motivations such as the change of the prescribed medications, a bad reaction to the medication and the lack of need to take this medication and these are among other reasons indicated by the respondents (Supplemental Figure S2.7a).

According to Table 3, gender (*p* = 0.01), age (*p* = 0.009) and distance from the pharmacy to the residence (*p* = 6.96 $\times 10^{-8}$ ) are the variables that mostly affect the disposal practices. Women are more associated with correct disposal practices, probably because they are usually responsible for managing the family’s medications. This is in line with the social context of Portugal, in which women are mostly responsible for domestic and family tasks. Regarding age, people aged 20–25 years are more likely to discard drugs in household waste. The distance to the pharmacy is another factor that influences the disposal method. More than half of the respondents (57%) live at a distance from the pharmacy equal or superior to 500 m (Supplemental Figure S2.3b). People who live between 200 and 500 m from the pharmacy are most likely to take the end-of-use pharmaceuticals to the pharmacy.

In the study of Shaaban et al. (2018), it was found that respondents having higher level of education or access to information are also more likely to return pharmaceutical waste to the pharmacy. This was not verified in our work, probably because the vast majority of the surveyed sample (77%) has higher school level of education.

### Environmental awareness for the adequate end-of-life management paths

To evaluate the public opinion of the Portuguese end-of-life system management practices of pharmaceuticals, a question on whether the delivery to pharmacies is encouraged was included in the survey. A total of 40% of the respondents considers that the current system promotes the delivery of pharmaceutical products to pharmacies, 25% have an opposite opinion and 35% believe that there could be other alternatives or incentives to an appropriate end of life for medicines (Supplemental Figure S2.7b). These results suggest that despite the current practices in Portuguese pharmacies, most people consider that there are still measures that could be taken to promote appropriate practices in the disposal of household medicines.

The survey lists several potential measures to promote the correct management of pharmaceuticals at their end of use: obtain a reward based on the number of pharmaceutical products delivered to the pharmacy; sensitize the population to the existing structures in terms of medication and packaging management and educate/train the public in guiding the correct rejection of discontinued medication; promote the dissemination of the activities of the management entity of empty packaging waste and discarded pharmaceutical products (VALORMED) through media strategies; and initiatives to be taken by the national government with goals and structures necessary to increase the collection of medicines. The first measure suggested is to offer small rewards to encourage citizens to return their end-of-life medicines to the pharmacy. These rewards could be product samples, discount vouchers, free blood pressure measurements, etc. Considering the possible lack of knowledge about the correct management of out-of-use medicines and packaging, the second measure aims to educate the population through school lectures and campaigns/events alluding to the problem. The third approach consists of disseminating information through social media. These are the measures that result in an approach to a large part of the population. The last measure suggests the development of a strategic plan by the national government to increase/improve medicine collection points. This action proposes to encourage the delivery of out-of-use medicines, since there are citizens who live considerably far from pharmacies.

The measures considered most relevant by the respondents included raising public awareness about existing structures responsible for the management of pharmaceutical residues; educating and training the public in guiding the correct rejection of pharmaceuticals. Respondents consider that raising awareness of the population on this problem would be the best approach to be taken. These measures would thus be possible to adopt to alert the population to the incorrect disposal of medicines, which may endanger both the environment and the public health. On the other hand, the possibility of receiving a reward in agreement with the number of deliveries to the pharmacy (e.g. as free samples of cosmetic and hygienic products, nutrition advice) was comparatively considered a less important solution (Table 4).

**Table 4.** Measures to increase the correct management of pharmaceutical products at the end of use by the population.

Measures	Degree of importance given by respondents (1 – least important; 5 – most important)				
	1	2	3	4	5
1. Obtain a reward based on the number of pharmaceutical products delivered to the pharmacy.	18.1%	13.0%	20.1%	21.5%	27.3%
2. Sensitize the population to the existing structures in terms of medication and packaging management and educate/train in guiding the correct rejection of discontinued medication.	3.6%	9.4%	10.9%	28.7%	47.4%
3. Promote the dissemination of the activities of the management entity of empty packaging waste and discarded pharmaceutical products (VALORMED) through media strategies (television, Facebook and Instagram).	3.8%	8.1%	16.3%	34.4%	37.4%
4. Initiatives to be taken by the national government (e.g. strategic plan for discontinued pharmaceutical products) with goals and structures necessary to increase the collection of medicines.	4.9%	10.3%	16.8%	32.0%	36.0%

It was possible to verify that most respondents considered the proposed measures as important or very important (Evaluation of 4 or 5).

**Figure 4.**
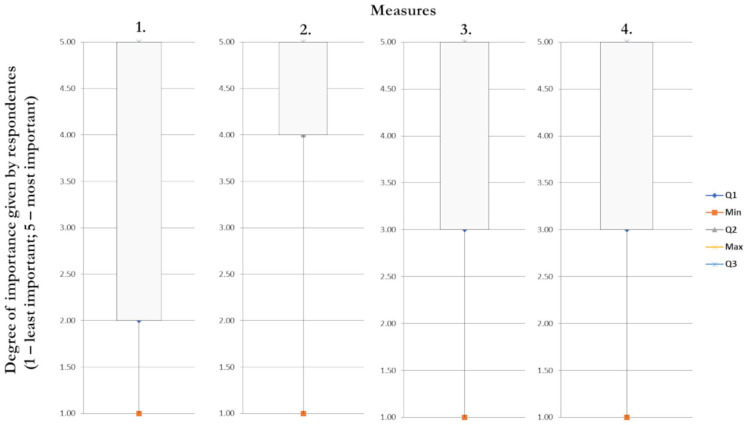
Boxplot with data concerning the measures to increase the correct management of pharmaceutical products at the end of use by the population.

Applying descriptive statistics though boxplots allowed to verify that while for measure 2, 75% of the respondents gave a rating equal or greater than 4, for questions 3 and 4 the same proportion of respondents gave a rating between 3 and 5. Nevertheless, 50% of the responses have classification between 4 and 5. Measure 1 showed the most variation in the assignment of a score (Figure 4).

On the one hand, the revision of published works with the same scope allows us to compare the practices among different countries, as well as the evolution of these practices over the years. This is essential to identify the measures that promote adequate pharmaceutical disposal, in order to implement them in other contexts. On the other hand, the study allows to understand the current practices at the Portuguese households, contributing to the state of the art. Furthermore, the recognition of the factors that most influence the disposal practices of pharmaceuticals assists in the development of focused and specific sustainable strategies. The results collected from the surveys carried out over the years reveal an improvement in pharmaceutical managements and a greater awareness of the environmental risks associated with inadequate management practices. This is largely due to increase in the access to information, the inclusion of this subject as research topic and the existence of awareness campaigns. Nevertheless, the lack of specific management plans for waste management is still an issue not to be neglected.

## Concluding remarks

The pharmaceutical industry has grown exponentially over the years and this trend is expected to continue. In an aging population, where the average life expectancy has been increasing and where chronic conditions are more and more frequent, the consumption of pharmaceutical products is part of the daily lives of families around the world. Despite the fact that this growth has indicatively contributed to the improvement of the quality of life, a major problem has been accentuated: the incorrect disposal of medicines.

In the present work, the addressed topic is contextualized by first associating with the growth of the pharmaceutical industry with increased risks to the environment and to human health. Thus, it is essential to promote the adequate disposal of pharmaceuticals used in the households in order to reduce these negative impacts. There are already several studies, summarized in this article, that sought to understand the common practices for pharmaceutical disposal. Through the reviewed studies, it was possible to conclude that there are still many households that do not deliver the end-of-use pharmaceuticals to the pharmacies, and the household trash is the most common disposal method. In this perspective, it is possible to consider that Portugal is one of the few countries where the percentage of people reporting to deliver pharmaceuticals to the pharmacy is higher (72%). While this is a good indicator, government efforts should continue to focus on measures to reduce the impacts generated by pharmaceutical products.

The assessment of the public opinion on this topic was also gathered. In other words, to understand the relevance that people attribute to the correct disposal of end-of-use pharmaceuticals, as well as their opinion about the current system adopted in Portugal, the survey informed about whether the existing system encourages citizen participation and about sensitivity measures that could be implemented to improve the management of end-of-use pharmaceuticals. While in Portugal, in order to deliver pharmaceutical waste, citizens have to go to pharmacies or to parapharmacies, there are countries that offer a larger variety of collection sites. In other countries, such as Poland, places where the respondents can bring wasted pharmaceuticals also include food shops, petrol station and via the Internet (at an online pharmacy or in response to an advertisement) (Rogowska et al., 2019). This shows that there are other practices to be adopted in other countries and emphasizes the importance of carrying out studies of this nature in order to understand the potential measures.

Other aspect that should be taken into consideration is the design of the medicine packages in a way that improve dismantling and sorting efficiency at its end of life. Nowadays, there is still a gap between the pharmaceutical industry and the use of life-cycle assessment (LCA) (Sousa et al., 2020). Despite the recognition by the pharmaceutical companies of the value of LCA towards more sustainable production and design, its use remains far from being a common practice (Emara et al., 2018). One reason for this may be the fact that the use of LCA tools is time-consuming and complex, but is also due to the lack of population awareness and laws and policies towards more sustainable paths for the waste management of unused pharmaceuticals (Mata et al., 2012).

Other crucial concept in environmental-based studies is the circular economy. Closing of the materials cycle is a leading principle within the circular economy strategy. In complement, the reduction of, or the efficient use of, raw materials may also well support the achievement of sustainability of processes and the implementation of either the materials closing (circularity) principle or the efficiency of the use of resources is undoubtedly relevant to also the pharmaceutical industry. Results obtained show that none of these (materials closing and resource efficiency) are being looked at. In fact, by contrast, the preferred pathways that households follow to dispose the medicines are far away from sustainable paths. A long way is still needed to perform to be able to maximize the medicines value and enable more sustainable practices within medicines supply chain (by increasing circularity).

In summary, the main outcomes of this study were as follows: (1) according to the studies worldwide, the most common method of disposal of end-of-use pharmaceuticals appears to be the household waste; (2) the majority of respondents in Portugal demonstrated a good awareness of the correct management of pharmaceutical waste, when compared to the reviewed studies; (3) most respondents consider that educating the population about the existing structures for managing pharmaceuticals and packaging is of high importance in improving the national management system and (4) gender, age and distance from the residence to the pharmacy have a significant contribution to the practices adopted (*p* < 0.05).

Although this is not the first national study for Portugal, studies carried out in this area are still very scarce and data are rarely updated. This is the first study that, in addition to updating the national reality, includes a statistical analysis of the data (that shows how gender, age and distance from the residence to the pharmacy affect the disposal methods). It also suggests concrete measures to promote adequate disposal ways and analyse the public opinion on disposal ways, and also presents an extensive analysis of the results obtained in other countries. Such findings will provide insights when creating future interventions to promote specific measures to enhance knowledge, change attitude and improve practice regarding disposal offend of life pharmaceutical products.

## Supplemental Material

sj-docx-1-wmr-10.1177_0734242X221105416 – Supplemental material for End-of-life management strategies of pharmaceuticals in Portuguese households Click here for additional data file. Supplemental material, sj-docx-1-wmr-10.1177_0734242X221105416 for End-of-life management strategies of pharmaceuticals in Portuguese households by Anabela Veiga, Ana Catarina Sousa, Cláudia Sousa, Manuel Oliveira and Belmira Neto in Waste Management & Research
