# Supplementary material for: End-of-life management strategies of pharmaceuticals in Portuguese households
Source: Waste Manag Res. 2022 Jul 27;41(1):235–47. doi: 10.1177/0734242X221105416 (PMC9773154; doi:10.1177/0734242X221105416)
Supplement: sj-docx-1-wmr-10.1177_0734242X221105416 – Supplemental material for End-of-life management strategies of pharmaceuticals in Portuguese households [file sj-docx-1-wmr-10.1177_0734242X221105416.docx]

**Supplementary Information**

**Initial survey information**: As part of a research project, we intend to analyse the management practices of end-of-use pharmaceuticals/medicines in Portuguese households. Consider as end-of-life all the medicines you do not intend to use (within or outside their expiry date). Please answer the following questionnaire only once and in an honest and thoughtful way. To aid the completion of this survey consider that medicines are: "substances or compositions of substances having properties for curing or preventing disease and its symptoms, in man or animal, with a view to establishing a medical diagnosis or to restoring, correcting or modifying its functions", INFARMED (2019). All answers are treated confidentially. Thank you very much for your participation.

**Survey - S1**

Gender:

- Feminine
- Masculine

Age:

- ≤ 20 years old
- 21 - 25 years old
- 26 - 30 years old
- 31 - 40 years old
- 41 - 60 years old
- ≥ 60 years old

Level of education:

- Incomplete elementary school
- Elementary school
- Middle school
- Higher education
- Professional technical courses

Situation regarding the respondents' work:

- Working
- Unemployed
- Housework
- Student
- Retired

Marital status:

- Single
- Married
- Divorced
- Separate
- Widow/widower

Residence (district):

- Aveiro
- Açores
- Beja
- Braga
- Bragança
- Castelo Branco
- Coimbra
- Évora
- Faro
- Guarda
- Leiria
- Lisboa
- Madeira
- Portalegre
- Porto
- Santarém
- Setúbal
- Viana do Castelo
- Vila Real
- Viseu
- Other

Household (including respondent):

- 1
- 2
- 3
- 4
- 5
- More than 5

Approximate distance from your usual pharmacy to your residence:

- < 200 m
- 200 to 500 m
- 500m to 1km
- > 1km

Indicate the number of different pharmaceutical product packages that you have in home (Note: count all packages, namely, those of the same medicine but of different strength or size).

- 0
- 1-5
- 6-10
- 11-20
- 21-30
- 31-40
- > 40

What kind of medicines do you usually have in your home? (you can choose more than one option)

- Analgesics, anti-inflammatory drugs (ben-u-ron, aspirin)
- Anxiolytics (to decrease anxiety and tension) and antiarrhythmics
- Antiasthmatic drugs
- Antibiotics (for example, penicillin derivatives)
- Antidepressants
- Antidiabetic drugs
- Antiepileptic drugs (anticonvulsant)
- Antihypertensive drugs (regulator of blood pressure)
- Antihistamines (to treat allergic reactions)
- Antipyretics (medicine that prevents or reduces fever) and antivirals
- Contraceptives
- Lipid regulators (for cholesterol)
- Treatment of acne (isotretinoin)
- Others

How do you dispose your medications?

- Separates the medicine from the packaging (cardboard box and leaflet).
- I don't do the separation.

What is the usual destination you give to out-of-use or term-based medications in your home?

Note: If you have not discarded the medication in the past few years, consider for answer what was done in your household the last time you discarded it. You can choose more than one option.

- Delivery at a pharmacy.
- Deposits in sewer / sink / toilet.
- Deposits in household waste.
- Others.

If you answered that you deliver your medicines to the pharmacy, select the most correct option.

- Take advantage of the moment of purchase to deliver your medicines out of use.
- You purposely go to the pharmacy to deliver your medicines out of use.
- I do not deliver medicines out of use in pharmacies

If you replied that you are delivering your medicines to the pharmacy, please indicate who is the person in the household responsible.

- Mother
- Father
- Son/daughter
- Grandfather
- Grandmother
- Maid
- Other
- I do not deliver medicines out of use in pharmacies.

How long has it been since you give a destination to the out-of-use medicines that exist in your home?

- ≤ 1 year
- 2 years
- 3 - 5 years
- > 5 years

What is the main reason why you discard out-of-use pharmaceutical products?

- The packaging was too big
- Stopped the medication / did not get along with that medication
- Changed medication/ change in medical prescription
- It's out of date
- You no longer need to take this medicine
- Another reason

Do you consider that the current system for delivering medicines that are no longer used in pharmacies promotes citizen participation?

- Yes
- No
- There could be other alternatives

Indicate in your opinion what is the importance for each of the measures to raise awareness of the correct management of medicines at the end of use? (1 - less important and 5 - more important).

- Obtain a reward based on the number of pharmaceutical products delivered to the pharmacy.
- Sensitize the population to the existing structures in terms of medication and packaging management and educate/train in the correct rejection of discontinued medication.
- Promote the dissemination of the activities of the management entity of empty packaging waste and discarded pharmaceutical products (VALORMED) through media strategies (Television, Facebook and Instagram).
- Initiatives to be taken by the national government (example strategic plan for discontinued pharmaceutical products) with goals and structures necessary to increase the collection of medicines.

**Data from respondents - S2**


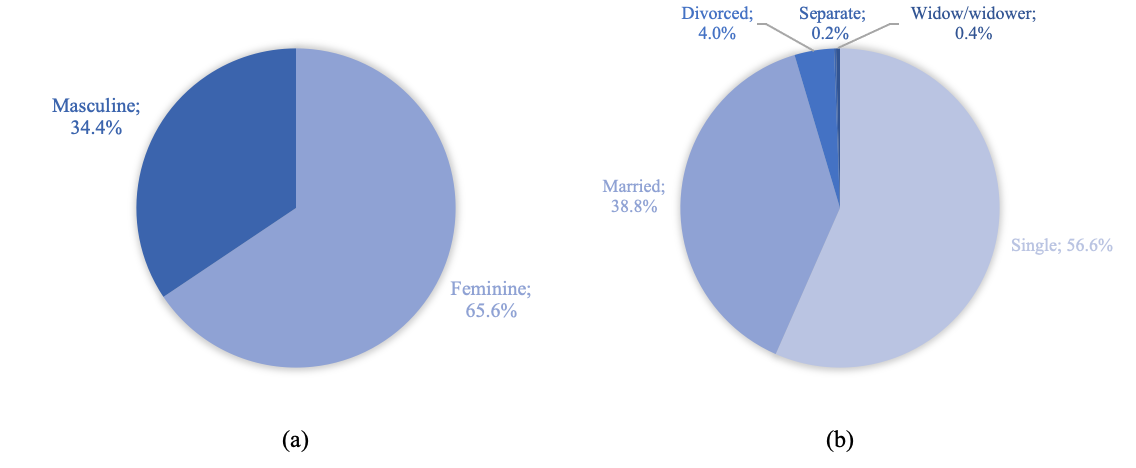


Figure S2.1: Characteristics of respondents: (a) gender and (b) marital status.

Figure S2.2: Characteristic of respondents: Residence (district).


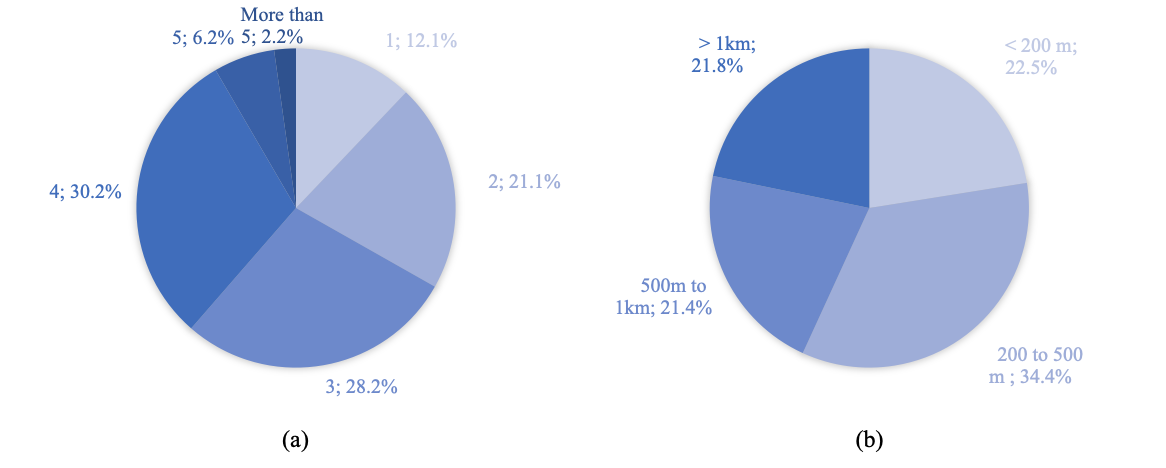


Figure S2.3: Data from respondents: (a) number of people living at this household and (b) approximate distance from your usual pharmacy to the residence.


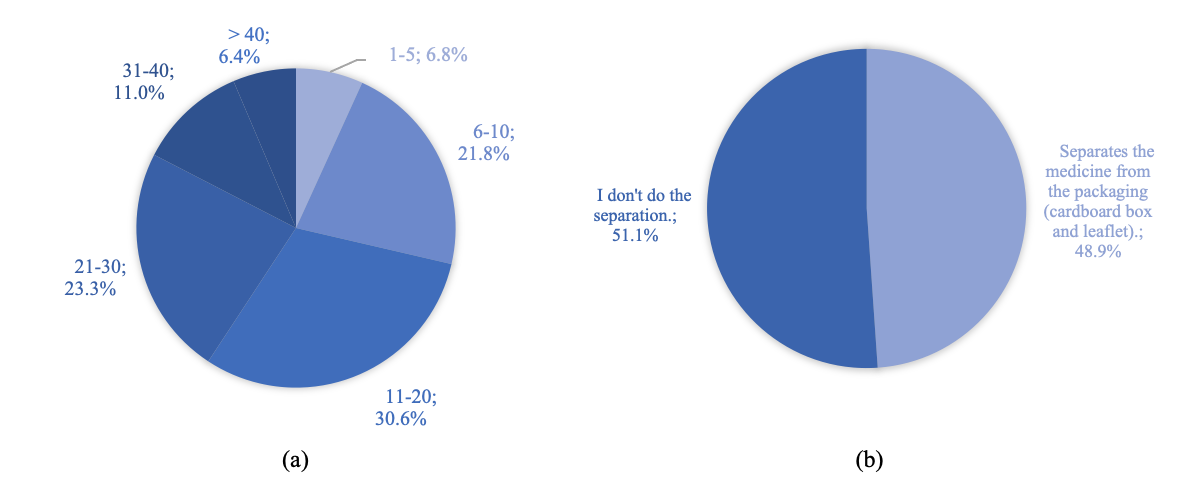


Figure S2.4: Data from respondents: (a) Number of different drug packages at home and (b) How do they dispose the medicines


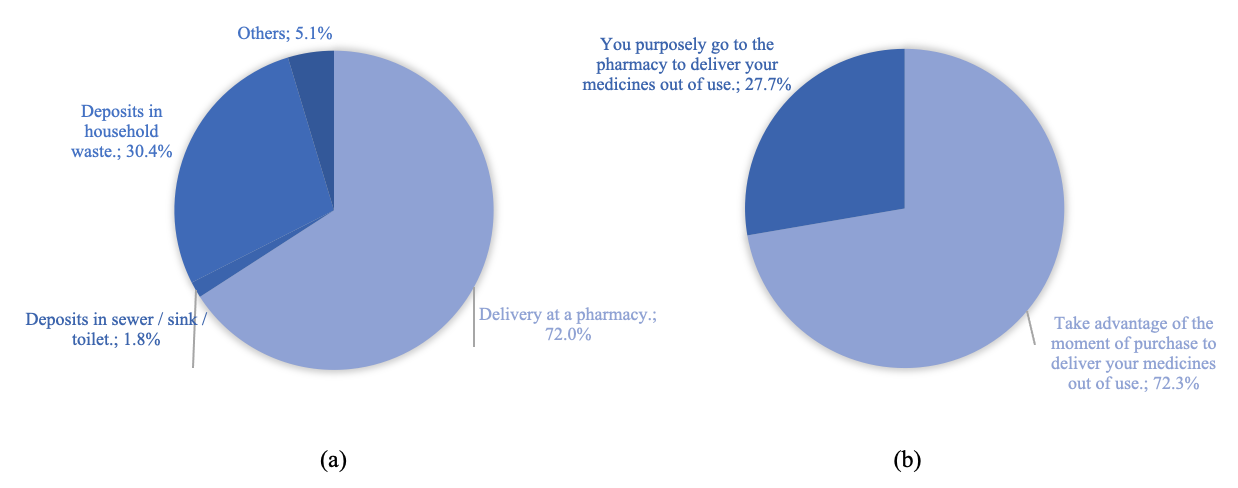


Figure S2.5: Data from respondents: In which situations the medication is disposed of.


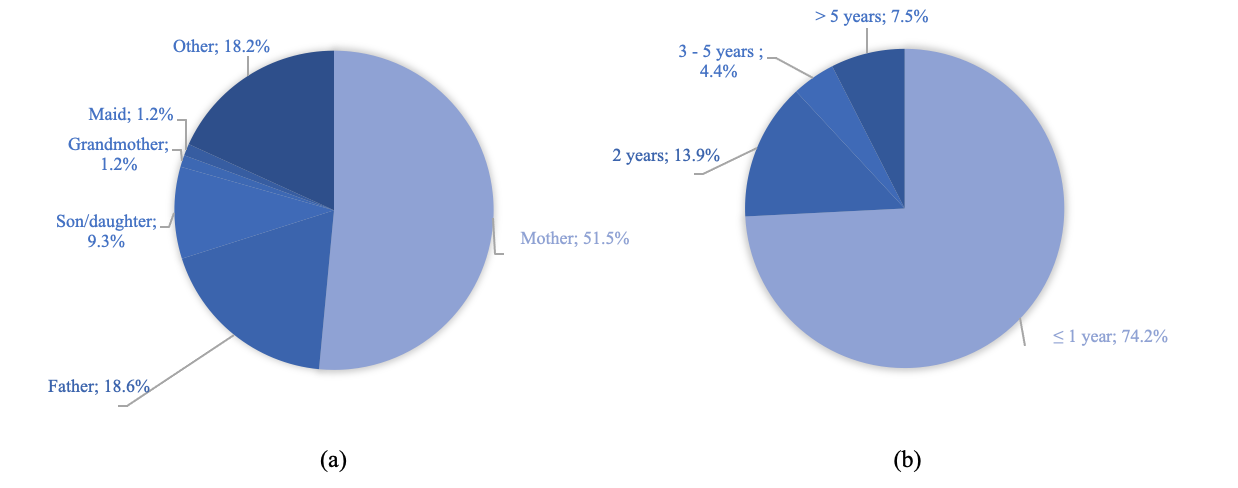


Figure S2.6: Data from respondents: (a) Who is the person in the household who discards the medication and (b) How long has it been since the respondent has not given a destination to medicines that are out of use.


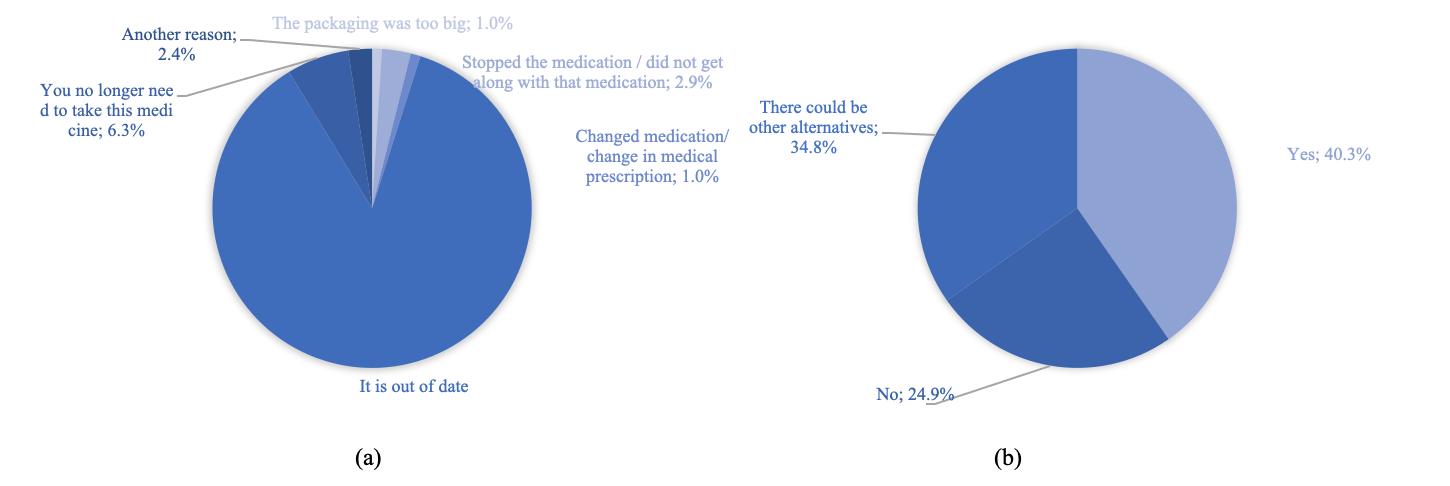


Figure S2.7: Data from respondents: (a) The main reason for disposing of medicines out of use and (b) If the current system of delivery of medicines out of use in pharmacies fosters citizen participation.
